# Systemic quinolones and risk of retinal detachment III: a nested case–control study using a US electronic health records database

**DOI:** 10.1007/s00228-021-03260-4

**Published:** 2022-03-15

**Authors:** Mohamed Kadry Taher, James A. G. Crispo, Yannick Fortin, Ryan Moog, Douglas McNair, Lise M. Bjerre, Franco Momoli, Donald Mattison, Daniel Krewski

**Affiliations:** 1 McLaughlin Centre for Population Health Risk Assessment, Faculty of Medicine, University of Ottawa, Room 216, 600 Peter Morand Crescent, Ottawa, ON K1G 5Z3 Canada; 2 School of Epidemiology and Public Health, University of Ottawa, Ottawa, ON Canada; 3 Risk Sciences International, Ottawa, ON Canada; 4 Department of Neurology, University of Pennsylvania Perelman School of Medicine, Philadelphia, PA USA; 5 Human Sciences Division, Northern Ontario School of Medicine, Sudbury, ON Canada; 6 Statistics Canada, Ottawa, ON Canada; 7 Cerner Corporation, Kansas City, MO USA; 8 Melinda Gates Foundation, Seattle, WA USA; 9 Children’s Hospital of Eastern Ontario Research Institute, Ottawa, ON Canada; 10 Department of Family Medicine, University of Ottawa, Ottawa, ON Canada; 11 Institut du Savoir Montfort, Ottawa, ON Canada

**Keywords:** Drug safety, Electronic health records, Nested case–control study, Pharmacovigilance, Quinolones, Retinal detachment

## Abstract

**Background:**

Quinolones are popular antibiotics that are known for their potency, broad coverage, and reasonable safety. Concerns have been raised about a possible association between quinolones and retinal detachment (RD).

**Methods:**

We conducted a nested case–control study using electronic health records (EHR) from the Health Facts® Database. The initial cohort included all patients who were admitted between 2000 and 2016, with no history of eye disease, and had a minimum medical history of one year. Eligible cases comprised inpatients who were first admitted with a primary diagnosis of RD between 2010 and 2015. Each eligible case was matched without replacement to five unique controls by sex, race, age, and period-at-risk. We used conditional logistic regression to calculate RD risk, adjusting for exposure to other medications, and major risk factors.

**Results:**

We identified 772 cases and 3860 controls. Whereas our primary analysis of all subjects revealed no quinolone-associated RD risk, elevated but non-significant risks were noted in African Americans (ciprofloxacin and levofloxacin), those aged 56–70 years old (moxifloxacin), and women (ciprofloxacin).

**Conclusion:**

Our study did not identify an elevated RD risk within 30 days following systemic administration of quinolone antibiotics. Suggestions of increased risk observed in some population subgroups warrant further investigation.

**Supplementary Information:**

The online version contains supplementary material available at 10.1007/s00228-021-03260-4.

## Introduction

Quinolones are a popular class of antibiotics that are heavily prescribed worldwide due to their potency, broad coverage, and reasonable safety [1–12]. Known adverse reactions to quinolones are mainly mild to moderate and self-limiting, although some quinolones have caused serious safety concerns, resulting in either revised labeling or market withdrawal [7–16]. The generous prescription of quinolones, among other antibiotics, is associated with a proportional increase in the emergence of quinolone-resistant bacterial strains [17–22]. However, despite the presence of other alternatives, quinolones continue to maintain their unique status as preferred treatments for a wide range of bacterial infections.

Retinal detachment (RD) is a serious medical condition with an annual incidence of 5–14/100,000 as reported from population studies conducted in Sweden, Finland, Croatia, Japan, and USA [23–25]. This involves the creation of breaks in the retinal layer with or without separation from its underlying tissues, with the subsequent loss of blood and oxygen supply, which requires urgent medical attention to avoid loss of vision [24, 26]. A possible mechanism for quinolone involvement in RD involves their ability to destroy the collagen content of the vitreous, with the resulting separation of the retina from the underlying tissues, a mechanism that resembles their damaging effect on collagen and connective tissues in joints and muscles, which led to a class-warning for possible tendon rupture [24, 27–33].

Many epidemiologic studies have examined quinolone-associated RD risk, with conflicting results [24, 33–49]. In 2013, the US FDA^1^ flagged a safety signal for quinolone-associated RD risk based on spontaneous reports to FAERS^2^ [38, 50]. However, in a 2017 drug safety communication, FDA reported on a lack of evidence of an increased RD risk due to quinolones [51]. Similarly, in 2016, Health Canada concluded that the evidence is insufficient to rule out such an association [52].

This study comprises one of a comprehensive, three-part examination of the association between systemic quinolones and risk of RD, which has been conducted in response to the heightened scientific and regulators’ safety concerns. The first part examines evidence from the FDA adverse drug event reporting system (FAERS) [53] to flag any disproportionality in voluntary reporting of possible quinolone-linked RD incidents beyond what would be normally expected (hypothesis generating). The verification of this association (hypothesis testing) is accomplished in the second part [54] (involving an analysis of clinical trial data) and the third part (current study), which examines data from a major database that includes electronic health records (EHR) of inpatients of more than 500 hospitals in the USA.

## Methods

### Data source

In this study, we analyzed inpatient EHR data from the Cerner Corporation’s Health Facts Data Warehouse® (Health Facts®), Kansas City, Missouri, USA. This large database includes detailed EHR for almost 70 million deidentified patients (approximately 21.6% of the US population^3^) that were generated between 2000 and 2016 via nearly 450 million encounters from more than 500 US hospitals. Health Facts® contains detailed patient information such as demographics, extensive medical care details, health care setting, and insurance status. Information on the number of cases, and the specific ICD9^4^ and ICD10^5^ codes used to identify RD cases, is provided in the Supplementary Material I and II, respectively. All eye diseases leading to exclusion of cases or controls from our study are defined in Supplementary Material III.

### Identification of cases and matched controls

Our three-part investigation of the association between quinolones and risk of RD focuses on acute onset RD in persons with otherwise healthy eyes. As acute onset involves RD with less than 2-week duration [55, 56], and given that a typical quinolone/antibiotic treatment would last 1–2 weeks, we restricted the duration of exposure assessment to 30 days prior to the de novo diagnosis of RD in persons with no current or prior eye diseases.

We identified an initial cohort comprising all inpatients who were admitted to any of the Health Facts® participating hospitals with no history of eye disease^6^ during the period 2000–2016. To allow for a comprehensive assessment of comorbidity of cases and controls, we excluded all patients with a medical history of less than 1 year in order to properly characterize the health status of study participants. To ensure consistency in the control matching process, we removed all patients with missing or inconsistent information on any of the matching variables (sex, race, and age at index encounter).

Finally, we restricted our cohort to inpatients for whom the date of index encounter was between 2010 and 2015, as preliminary exploration of the database revealed very sparse reporting of RD prior to that period. The index date for a case represents the date of the first encounter where a patient was admitted with a primary RD diagnosis; for a control, this date represents the date of the latest inpatient encounter without being diagnosed with RD. We calculated the period-at-risk, which represents the time interval between date of the first recorded inpatient encounter and date of the index encounter, for both cases and controls.

An optimal variable matching approach [57] was used for matching controls to cases without replacement, where each control was matched to a single case. Each case was matched to five controls based on four variables with equal weights: age on day of the index encounter (± 1 year), sex, race, and the period-at-risk (± 1 year).

### Medication exposure

As we are interested in systemic quinolones, all non-systemic formulations were excluded. Inpatient medication exposure was grouped into three classes: quinolones, other antibiotics (excluding quinolones), and all other medications combined (excluding antibiotics). Data on medication exposure was limited to prescriptions filled during inpatient care.

### Data analyses

#### Descriptive analyses

We reported categorical variables as frequencies and percentages, and continuous variables as means with standard deviations. Categorical variables included sex, race (Caucasian, African American, Asian, Hispanic, other), socioeconomic indicators including census region and division, hospital setting (urban/rural); health insurance (insured, non-insured, missing/unknown), and 30-day exposure to each of the three medication groups and individual quinolones (ever/never). Additional variables included diabetes mellitus (complicated; ever/never) and alcohol abuse.

Continuous variables included age at index encounter, comorbidity status, and the number of medications filled during the 30-day period preceding the index encounter. Age was stratified into 10-year intervals (0–10, 11–20, 21–30, 31–40, 41–50, 51–60, 61–70, 71–80, and 81–90 years). Comorbidity was measured by the score generated via the Hude Quan version [58] of the Elixhauser comorbidity index (CMI) [59], stratified into 5 categories (CMI = 0, 1–5, 6–10, 11–15, and 16 +). The number of inpatient medication prescriptions was stratified into five groups (0, 1–3, 4–6, 7–10, and 11 +).

#### Regression analyses

We generated a series of conditional logistic regression models to identify the best estimate of quinolone-associated RD risk, while adjusting for other medication exposures and major confounders. For each medication group, we fitted a base model including only sex, race, age at index encounter as matching variables, and use (ever/never) of the medication group as the independent variable. We then fit a minimally adjusted model including all variables in the base model as well as all other medication groups (ever/ never). Finally, we fit a maximally adjusted model, extending the minimally adjusted model to include health insurance, census division, hospital setting/type, diabetes mellitus, and alcohol abuse, as potentially important demographic and socioeconomic covariates. To identify the individual quinolone(s) with the strongest possible association with RD risk, we repeated the same series of regression models using exposure to individual quinolones, rather than a class, as predictors of RD.

To avoid possible confounding, we excluded a priori all patients with a history of eye diseases. We also adjusted for other major confounders, including health status, major risk factors (diabetes mellitus and alcohol abuse), and socioeconomic status (health insurance and care setting).

#### Subgroup analyses

To isolate the effect of notable differences in comorbidity and inpatient medication prescribing between cases and controls, we fitted a third series of regression models to different subgroups of our study population via stratifying by sex, race, comorbidity status (tertiles), and age at index encounter (tertiles).

## Results

### Identification of RD cases

The entire Health Facts® Data Warehouse contained unique patients who were admitted at least once between 2000 and 2016 to any of the Health Facts® participating hospitals. By excluding inpatients with prior eye diseases, we identified 67,117,520 potentially eligible patients. By removing all patients with a medical history of less than 1 year, we were able to identify an initial study cohort of 3,361,592 individuals.

**Fig. 1:**
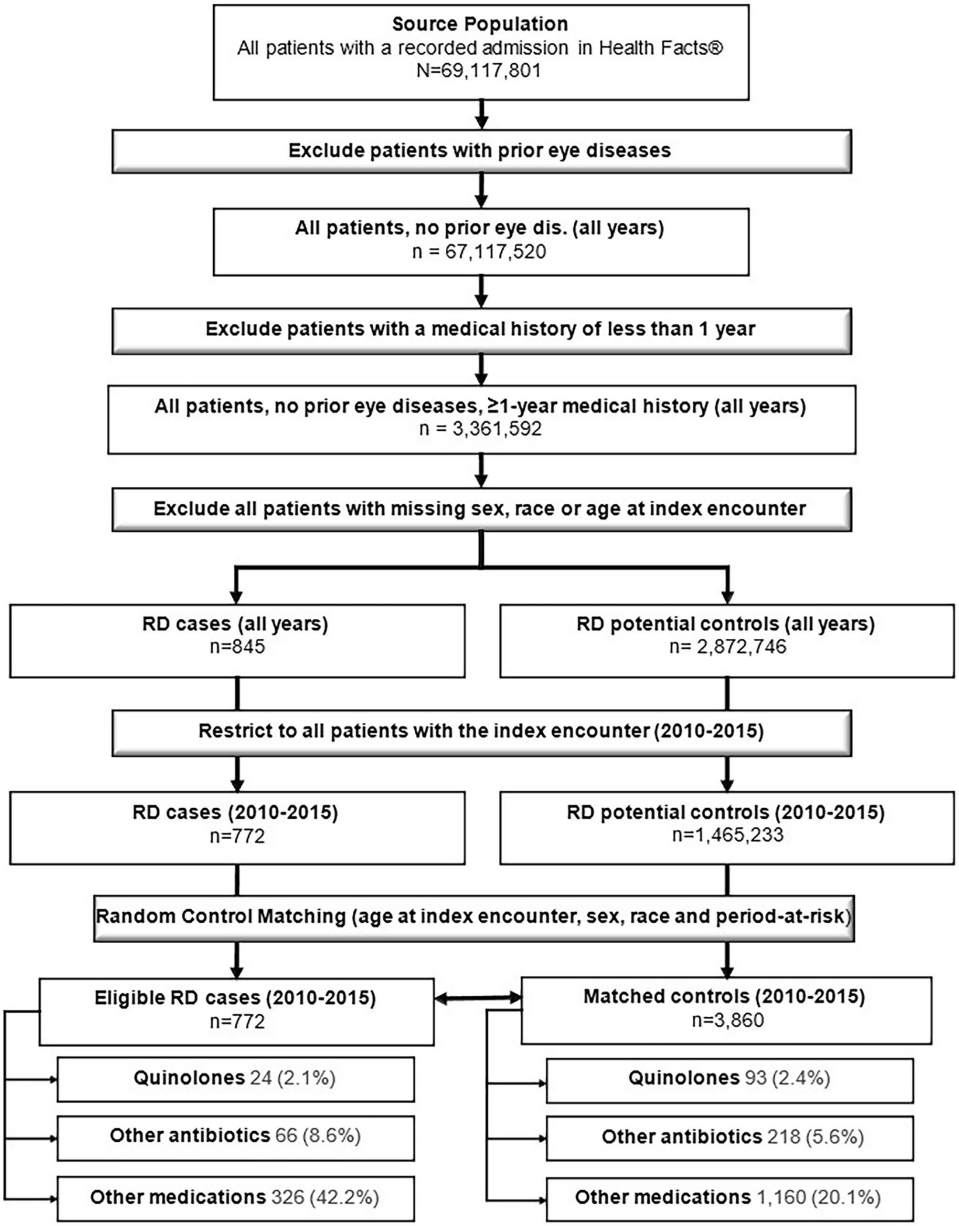
Identification of eligible RD cases and matching controls (2010–2015)

Excluding those with missing or inconsistent data for any of the matching variables restricted this pool to 2,873,591 subjects, which included 845 RD cases and 2,872,746 potential controls. We then removed cases and potential controls with an index date between 2010 and 2015 to reach a final cohort consisting of 772 cases and 1,465,233 potential controls. Based on our matching algorithm, we were able to match a total of 3860 unique controls to the 772 cases (see Fig. 1).

### Characteristics of study population

The final study population was predominantly Caucasian (77.6%) and included slightly more men (51%) than women. Incidence of RD ranged from 3 to 5% in the first four decades of life, and doubled twice to 9.3% and 21% in the fifth and sixth decades, respectively, before reaching a plateau afterward. Whereas there were more controls than cases with no comorbidities (34% compared to 15%), cases with comorbidities showed consistently higher comorbidities than controls, particularly within the CMI level 1–5 (55% compared to 43%). A higher prevalence of diabetes mellitus was identified in cases compared to controls at both levels of severity, uncomplicated (33% compared to 21%) and complicated (18% compared to 9%). Alcohol abuse showed no difference in prevalence between the two groups. Further details on the study population are shown in Table 1.

**Table 1:** Characteristics of cases and matched controls

Characteristics	No. (%) of patients or mean (± SD)		*p*-value
	**Cases**	**Controls**	
**Total no. of participants**	772	3860	
**Sex**^a^			**0.89**
Women	378 (49.0%)	1,879 (48.7%)	
Men	394 (51.0%)	1,981 (51.3%)	
**Race class**^**a**^			**0.98**
Caucasian	599 (77.6%)	3,004 (77.8%)	
African American	118 (15.3%)	589 (15.3%)	
Asian	9 (1.2%)	37 (1.0%)	
Hispanic	3 (0.4%)	18 (0.5%)	
Other	43 (5.6%)	212 (6.0%)	
**Age group**^a^			**1.0000**
0–10	46 (6.0%)	229 (5.9%)	
11–20	32 (4.2%)	161 (4.2%)	
21–30	22 (2.9%)	112 (2.9%)	
31–40	43 (5.6%)	213 (5.5%)	
41–50	72 (9.3%)	360 (9.3%)	
51–60	165 (21.4%)	825 (21.4%)	
61–0	174 (22.5%)	870 (22.5%)	
71–80	151 (19.6%)	756 (19.6%)	
81 +	67 (8.7%)	334 (8.7%)	
**Census region**			** < .0001**
South	244 (31.6%)	1113 (28.8%)	
North East	226 (29.3%)	538 (13.9%)	
Midwest	179 (23.2%)	1721 (44.6%)	
West	123 (15.9%)	488 (12.6%)	
**Hospital setting**			** < .0001**
Urban	624 (80.8%)	2694 (69.8%)	
Rural	148 (19.2%)	1166 (30.2%)	
**Health insurance**			**0.0096**
Insured	642 (83.2%)	3347 (86.7%)	
Non-insured	27 (3.5%)	139 (3.6%)	
Unknown/missing	103 (13.3%)	374 (9.7%)	
**Payer group**			**0.0143**
HMO/managed care	417 (54.0%)	2035 (52.7%)	
Free, research	225 (29.2%)	1309 (33.9%)	
Self-pay	26 (3.4%)	137 (3.6%)	
Other	1 (0.1%)	5 (0.1%)	
Unknown/missing	103 (13.3%)	374 (9.7%)	
**Comorbidity score**	**4.12 ( 3.57)**	**3.08 ( 3.7)**	** < .0001**
0	112 (14.5%)	1315 (34.1%)	
1–5	422 (54.7%)	1674 (43.4%)	
6–10	190 (24.6%)	660 (17.1%)	
11–15	44 (5.7%)	194 (5.0%)	
16 +	4 (0.52%)	17 (0.45%)	
**Confounders**			
Diabetes–uncomplicated	252 (32.6%)	824 (21.4%)	< .0001
Diabetes–complicated	140 (18.1%)	328 (8.5%)	< .0001
Alcohol abuse	36 (4.7%)	171 (4.4%)	0.7747

*HMO* Health Management Organizations

^**a**^Matching variables

The average number of quinolone prescriptions per patient filled during the 30 days preceding the index date was comparable between cases (0.044 ± 0.28) and controls (0.037 ± 0.26). However, prescribing of other non-quinolone medications was higher in cases compared to controls (see Supplementary Material IV). Each of ciprofloxacin, levofloxacin, and moxifloxacin were similarly prescribed among cases and controls during the 30 days preceding the index date, with moxifloxacin being prescribed only once for a single case and once for each of three controls (see Supplementary Material IV).

### Regression analysis

#### Entire study population

Our primary analysis of the entire study population showed that exposure to systemically administered quinolone antibiotics was not associated with an increased risk of RD [(aOR: 0.75 (95% CI: 0.43–1.32)], upon adjusting to exposure to non-quinolone antibiotics and other medications combined, as well as major risk factors for this outcome. Repeating the same analysis based on individual quinolones produced similar results to the multi-medication model including all quinolones simultaneously. The risk estimates reported in Table 2 for the two medication groups comprised quinolones and other non-quinolone antibiotics and in Table 3 for individual quinolones were generated using the multi-medication model.

**Table 2:** Base and adjusted odds ratios (aOR) for risk of retinal detachment with quinolones compared to non-quinolone antibiotics

Population and medication group	Base model^a^ OR (95% CI)	*p*-value	Maximally adjusted model^b^ aOR (95% CI)	*p*-value
**Entire population**				
Quinolones	1.27 (0.80–2.01)	0.3055	0.75 (0.43–1.32)	0.3184
Non-quinolone antibiotics	1.54 (1.15–2.06)	0.0036	0.86 (0.58–1.26)	0.4259
**Comorbidity level**				
***CMI:0–1***				
Quinolones	1.40 (0.25–7.79)	0.6984	0.70 (0.05–9.74)	0.7914
Non-quinolone antibiotics	2.66 (1.00–7.08)	0.0503	1.12 (0.28–4.44)	0.8777
***CMI:2–4***				
Quinolones	0.46 (0.09–2.29)	0.3420	0.35 (0.05–2.42)	0.2872
Non-quinolone antibiotics	0.62 (0.22–1.79)	0.3782	0.49 (0.12–1.92)	0.3053
***CMI:5* + **				
Quinolones	0.87 (0.45–1.66)	0.6624	0.49 (0.21–1.17)	0.1094
Non-quinolone antibiotics	1.46 (0.91–2.33)	0.1173	1.15 (0.55–2.38)	0.7155

^a^Base model: age, sex, race variables, and the tested medication group

^b^Maximally adjusted model: minimally adjusted, and complicated diabetes mellitus, alcohol abuse and socioeconomic status (census division, hospital (urban/rural), and insurance)

**Table 3:** Base and adjusted odds ratios (aOR) for risk of retinal detachment in relation to use of individual quinolones

Population and individual quinolone	Base model OR (95% CI)	*p*-value	Maximally adjusted model aOR (95% CI)	*p*-value
**Entire population**				
Ciprofloxacin	1.60 (0.83–3.07)	0.1588	0.87 (0.39–1.97)	0.7415
Levofloxacin	0.94 (0.48–1.81)	0.8411	0.61 (0.29–1.30)	0.1984
Moxifloxacin	1.67 (0.17–16.02)	0.6582	1.07 (0.10–11.08)	0.9535
**Comorbidity level**				
***CMI:0–1***				
Ciprofloxacin	2.78 (0.38–20.39)	0.3157	1.00 (0.05–19.93)	0.9987
Levofloxacin	< 0.001 (< 0.001– > 999.999)	0.9826	< 0.001 (< 0.001– > 999.999)	0.9905
Moxifloxacin	N/A	N/A	N/A	N/A
***CMI:2–4***				
Ciprofloxacin	0.40 (0.04–3.75)	0.4239	0.15 (0.01–2.05)	0.1546
Levofloxacin	0.54 (0.05–5.47)	0.5981	1.07 (0.08–13.78)	0.9577
Moxifloxacin	N/A	N/A	N/A	N/A
***CMI:5* + **				
Ciprofloxacin	1.24 (0.44–3.43)	0.6858	0.74 (0.19–2.91)	0.6606
Levofloxacin	0.70 (0.29–1.74)	0.4455	0.46 (0.16–1.39)	0.1691
Moxifloxacin	0.65 (0.07–6.60)	0.7190	0.14 (0.01–3.59)	0.2369

Upon stratifying the study population into tertiles (0–1, 2–4, 5 +) based on their comorbidity status (CMI score), neither quinolones nor other antibiotics showed any RD risk upon adjusting to major confounders. Quinolone antibiotics showed an almost twofold non-significant increase in RD risk in African Americans [aOR: 2.88 (95% CI: 0.43–19.33)]. This risk was driven by ciprofloxacin [aOR: 2.09 (95% CI: 0.11–41.09)] and levofloxacin [aOR: 1.85 (95% CI: 0.08–42.88)].

Whereas quinolones showed no difference in RD risk between men and women, ciprofloxacin showed a marginal, non-significant increased risk in women [aOR: 1.34 (95% CI: 0.34–5.22)]. Upon stratifying the study population by age into tertiles (0–55, 56–70, 71 + years), quinolones were not associated with an increased risk in any age group. However, moxifloxacin was associated with a more than fourfold, but highly uncertain, increase in RD risk in the (56–70) year-old group [aOR: 5.36 (95% CI: 0.30–97.21)].

Our primary analysis of the entire study population showed an increased RD risk in association with complicated diabetes mellitus [aOR: 2.19 (95% CI: 1.68–2.86)]. Upon stratifying the study population, a similar pattern was identified in population subgroups, particularly in the healthiest comorbidity tier (CMI: 0–1): 14.08 (95% CI: 1.40–141.93)]. An increased risk was also noted in women [aOR: 2.69 (1.82–4.00)] more so than in men [aOR: 1.82 (95% CI: 1.26–2.63)], and in African Americans [aOR: 5.85 (95% CI: 2.70–12.65)] more so than in Caucasians [aOR: 1.69 (95% CI: 1.24–2.30)]. A fivefold risk was also noted in the youngest age tertile (0–55): [aOR: 6.18 (95% CI: 3.65–10.46)], which declined and became non-significant in the older age tertiles. In contrast to diabetes, alcohol abuse showed no elevated RD risk in either the entire study population or population subgroups.

Results for the base and maximally adjusted models for the primary analysis examining the entire study population and the subgroup analysis based on comorbidity score are presented in this manuscript for all medication groups and for the individual quinolones in Tables 2 and 3, respectively. Complete listings of the ORs, 95% CI and *p*-value for all regression analyses are provided in Supplementary Material V-VII.

## Discussion

Examining our entire study population revealed no evidence of a quinolone class-wide association with increased RD risk within 30 days of administering a systemic preparation of a quinolone antibiotic. However, a nearly twofold non-significant increase in RD risk in African Americans was attributable to ciprofloxacin and levofloxacin. Moxifloxacin showed more than fourfold non-significant increase in RD risk in those 56–70 years of age. Ciprofloxacin showed also a marginal and non-significant increase in RD risk in women. An overall low consumption of medications, particularly antibiotics, reflected a relatively healthy population with minimal to moderate comorbidity burden.

Patients with complicated diabetes mellitus showed a consistently increased RD risk in all analyses. However, diagnosis of alcohol abuse showed an increased and non-significant RD risk only in Caucasians, women, and those ≥ 71 years of age. A complete listing of risk estimates for all analyses involving diabetes mellitus and alcohol abuse is provided in Supplementary Material VII.

Similar to an earlier study that utilized Health Facts® data for a different outcome, the total number of RD cases was remarkably low except, between 2010 and 2015 [60]. Prior to 2010, this may have been attributable to gradual enrollment into Health Facts®, whereas 2016 marked the adoption of the new ICD10 coding with a subsequent drop in the number of recorded cases. Accordingly, we used ton data only from 2010 to 2015 in the present analysis.

To avoid possible confounding of the association between quinolones and RD risk, we excluded all inpatients with current or prior eye diseases, and adjusted for two major risk factors, complicated diabetes mellitus and alcohol abuse. Since the study subjects were inpatients, we used medication filling orders as proxy for medication administration with a high degree of confidence that the medications were consumed by patients while in the hospital.

In our study, we selected a nested case–control rather than cohort design since the former offers similar benefits, but with greater computational efficiency than the cohort design [61–63]. The nested case–control design allows for matching on age and calendar time, rather than adjusting for these effects as covariates in a cox regression model widely used in the analysis of cohort data [61–63]. Missing observations may also have a lesser impact in a nested case–control analysis compared to the cohort design [64–66].

To put our results in context with those of recent major epidemiologic studies that examined the association of quinolones with RD risk, we identified eight original studies [24, 33–39], three systematic reviews [40–42], and one umbrella review [43]. Whereas all reviews [40–43] and five original studies [33–36, 38] reported no association between oral/systemic quinolone administration and RD risk, only three studies [24, 37, 39] reported an increased RD risk with use of quinolone antibiotics. These inconsistencies may be due to differences in factors such as study design, target population, and sampling frame (further details of these studies are provided in Supplementary Material VIII).

A major strength for our study is its use of a major EHR database, which provided a great opportunity for studying large patient populations over a long period of time, thereby supporting meaningful investigation of a rare adverse drug reaction such as RD [67, 68]. With the availability of comprehensive patient-related information such as demographics, diagnoses, clinical assessments, diagnostic, medical and surgical procedures, and patient outcomes, it was possible to assess the temporality of association of between exposure (quinolone antibiotics) and outcome (RD), adjusting for medication exposure and major risk factors [67–70].

Limiting our pool of cases and possible controls to those with a medical history of 1 year at a minimum allowed for a better assessment of the health status of the examined study population. Finally, using prescriptions filled for our study population and delivered by nursing staff as proxy for medication administration provided more confidence in patients’ medication compliance compared to medications prescribed on an outpatient basis.

Similar to other EHR databases, Health Facts® was primarily created for the purpose of supporting a seamless exchange of patients’ medical information among providers across the care continuum. However, despite all robust data cleaning techniques, EHR databases are subject to data integrity issues such as misclassification of demographics, comorbidities, medications, outcomes, or other clinical care details [71, 72]. Additionally, Health Facts® lacked information on outpatient or consumption of over-the-counter medication consumption, precluding a comprehensive examination of possible polypharmacy effects [70].

Considering the fact that 50–80% of antibiotics are prescribed in physician offices rather than hospitals [17, 73], records of antibiotic use in Health Facts® are necessarily incomplete. However, prescriptions filled during hospital stay may reflect outpatient prescription patterns to a large extent. If outpatient and inpatient prescribing patterns differed notably, this would lead to exposure misclassification, which, if random, would be expected to bias the risk estimates toward the null value of no effect.

## Conclusion

Our study results provided no evidence of an increased class-wide association between quinolones and RD risk. Care should be exercised in interpreting the results of this study due to small number of filled quinolone prescriptions during the 30-day window prior to case ascertainment. Further attention should be directed at quinolone exposure within certain population subgroups such as women, those 56–70 years old, and African Americans. Further studies with additional information on outpatient medication use would be also complement the present findings.

## Supplementary Information

Below is the link to the electronic supplementary material.

Supplementary file1 (PDF 140 KB)

## Data Availability

Data used in this study has been extracted from electronic health records of the Cerner Health Facts® database (Kansas City, Missouri, USA), which are securely stored and maintained by the McLaughlin Centre for Population Health Risk Assessment of the University of Ottawa (Ottawa, Ontario, Canada). External sharing of this data is not permissible.

## Footnotes

1 FDA: Food and Drug Administration.

2 FAERS: FDA Adverse Event Reporting System.

3 US population as of December 31, 2016: 324,070,652 (https://www.census.gov).

4 International Classification of Diseases, Ninth Revision (ICD-9).

5 International Classification of Diseases, Tenth Revision, Clinical Modification (ICD-10-CM).

6 Except for the outcome of interest, retinal detachment (RD).
