# Supplementary material for: Systemic quinolones and risk of retinal detachment III: a nested case–control study using a US electronic health records database
Source: Eur J Clin Pharmacol. 2022 Mar 15;78(6):1019–28. doi: 10.1007/s00228-021-03260-4 (PMC9107393; doi:10.1007/s00228-021-03260-4)
Supplement: Supplementary file 1 — Supplementary file1 (PDF 140 KB) [file 228_2021_3260_MOESM1_ESM.pdf]

Systemic Quinolones and Risk of Retinal Detachment III: A Nested Case-Control Study Using a US Electronic Health Records Database

Supplementary Material

Table of Contents

I. Annual recording of cases of retinal detachment (RD) ... 3

II. List of ICD-9/ICD-10 codes for identifying cases of retinal detachment (RD) ... 4

III. List of eye diseases leading to exclusion of cases ... 5

IV. Odds ratio and 95% CL for quinolones and other medication groups, and risk of retinal detachment ... 23

V. Odds ratio and 95% CL for individual quinolones and risk of retinal detachment 30

VI. Odds ratios and 95% CI for complicated diabetes mellitus and alcohol abuse, and risk of retinal detachment ... 34

VII. Results from recent studies on quinolone antibiotics and risk of retinal detachment 36

VIII. References ... 41

I. Annual recording of cases of retinal detachment (RD)

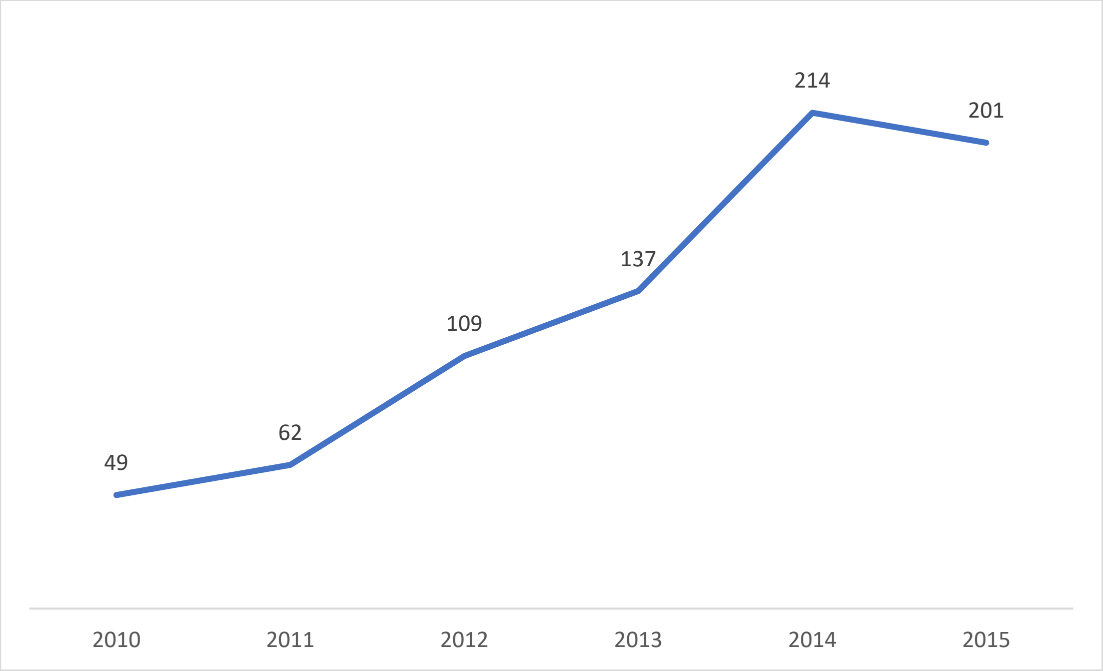

Figure 1: Final group of eligible RD cases, by year of diagnosis (2010-2015)

II. List of ICD-9/ICD-10 codes for identifying cases of retinal detachment (RD)

ICD Type	ICD Code	Details
ICD 9	361.03	Recent Retinal Detachment, Partial, with Giant Tear
	361.89	Other Forms of Retinal Detachment
	361	Retinal Detachment with Retinal Defect
	361	Retinal Detachment with Retinal Defect, Unspecified
	361.01	Recent Retinal Detachment, Partial, with Single Defect
	361.02	Recent Retinal Detachment, Partial, with Multiple Defects
	361.04	Recent Retinal Detachment, Partial, with Retinal Dialysis
	361.05	Recent Retinal Detachment, Total or Subtotal
	361.06	Old Retinal Detachment, Partial
	361.07	Old Retinal Detachment, Total or Subtotal
	361.2	Serous Retinal Detachment
	361.8	Other Forms of Retinal Detachment
	361.9	Unspecified Retinal Detachment
	361.03	Recent Retinal Detachment, Partial, with Giant Tear
ICD 10-CM	H33	Retinal detachments and breaks, Excl.: detachment of retinal pigment epithelium (H35.7)
	H33.0	Retinal detachment with retinal break (Rhegmatogenous retinal detachment)
	H33.1	Retinoschisis and retinal cysts
	H33.2	Serous retinal detachment
	H33.3	Retinal breaks without detachment

III. List of eye diseases leading to exclusion of cases

ICD type	ICD code	Details
ICD9	91.51	Syphilitic Chorioretinitis (Secondary)
ICD9	94.83	Syphilitic Disseminated Retinochoroiditis
ICD9	130.2	Chorioretinitis Due to Toxoplasmosis
ICD9	190	Malignant Neoplasm of Eyeball, Except Conjunctiva, Cornea, Retina, and Choroid
ICD9	190.5	Malignant Neoplasm of Retina
ICD9	224	Benign Neoplasm of Eyeball, Except Conjunctiva, Cornea, Retina, and Choroid
ICD9	224.5	Benign Neoplasm of Retina
ICD9	228.03	Hemangioma of Retina
ICD9	239.81	Neoplasm of Unspecified Nature of Retina and Choroid
ICD9	361	Retinal Detachments and Defects
ICD9	361.01	Recent Retinal Detachment, Partial, with Single Defect
ICD9	361.02	Recent Retinal Detachment, Partial, with Multiple Defects
ICD9	361.03	Recent Retinal Detachment, Partial, with Giant Tear
ICD9	361.04	Recent Retinal Detachment, Partial, with Retinal Dialysis
ICD9	361.05	Recent Retinal Detachment, Total or Subtotal
ICD9	361.06	Old Retinal Detachment, Partial
ICD9	361.07	Old Retinal Detachment, Total or Subtotal
ICD9	361.1	Retinoschisis and Retinal Cysts
ICD9	361.13	Primary Retinal Cysts
ICD9	361.14	Secondary Retinal Cysts

ICD type	ICD code	Details
ICD9	361.19	Other Retinoschisis and Retinal Cysts
ICD9	361.3	Retinal Defects without Detachment
ICD9	361.3	Retinal Defect, Unspecified
ICD9	361.31	Round Hole of Retina without Detachment
ICD9	361.32	Horseshoe Tear of Retina without Detachment
ICD9	361.33	Multiple Defects of Retina without Detachment
ICD9	361.81	Traction Detachment of Retina
ICD9	362	Other Retinal Disorders
ICD9	362.1	Other Background Retinopathy and Retinal Vascular Changes
ICD9	362.13	Changes in Vascular Appearance of Retina
ICD9	362.14	Retinal Microaneurysms Nos
ICD9	362.15	Retinal Telangiectasia
ICD9	362.16	Retinal Neovascularization Nos
ICD9	362.18	Retinal Vasculitis
ICD9	362.3	Retinal Vascular Occlusion
ICD9	362.3	Retinal Vascular Occlusion, Unspecified
ICD9	362.31	Central Retinal Artery Occlusion
ICD9	362.32	Retinal Arterial Branch Occlusion
ICD9	362.33	Partial Retinal Arterial Occlusion
ICD9	362.34	Transient Retinal Arterial Occlusion
ICD9	362.35	Central Retinal Vein Occlusion
ICD9	362.36	Venous Tributary (Branch) Occlusion of Retina
ICD9	362.37	Venous Engorgement of Retina

ICD type	ICD code	Details
ICD9	362.4	Separation of Retinal Layers
ICD9	362.4	Retinal Layer Separation, Unspecified
ICD9	362.42	Serous Detachment of Retinal Pigment Epithelium
ICD9	362.43	Hemorrhagic Detachment of Retinal Pigment Epithelium
ICD9	362.5	Degeneration of Macula and Posterior Pole of Retina
ICD9	362.5	Macular Degeneration (Senile) of Retina, Unspecified
ICD9	362.51	Nonexudative Senile Macular Degeneration of Retina
ICD9	362.52	Exudative Senile Macular Degeneration of Retina
ICD9	362.53	Cystoid Macular Degeneration of Retina
ICD9	362.54	Macular Cyst, Hole, or Pseudohole of Retina
ICD9	362.55	Toxic Maculopathy of Retina
ICD9	362.56	Macular Puckering of Retina
ICD9	362.57	Drusen (Degenerative) of Retina
ICD9	362.6	Peripheral Retinal Degenerations
ICD9	362.6	Peripheral Retinal Degeneration, Unspecified
ICD9	362.61	Paving Stone Degeneration of Retina
ICD9	362.62	Microcystoid Degeneration of Retina
ICD9	362.63	Lattice Degeneration of Retina
ICD9	362.64	Senile Reticular Degeneration of Retina
ICD9	362.65	Secondary Pigmentary Degeneration of Retina
ICD9	362.7	Hereditary Retinal Dystrophies
ICD9	362.7	Hereditary Retinal Dystrophy, Unspecified
ICD9	362.71	Retinal Dystrophy in Systemic or Cerebroretinal Lipidoses

ICD type	ICD code	Details
ICD9	362.72	Retinal Dystrophy in Other Systemic Disorders and Syndromes
ICD9	362.74	Pigmentary Retinal Dystrophy
ICD9	362.75	Other Dystrophies Primarily Involving the Sensory Retina
ICD9	362.76	Dystrophies Primarily Involving the Retinal Pigment Epithelium
ICD9	362.77	Retinal Dystrophies Primarily Involving Bruch's Membrane
ICD9	362.8	Other Retinal Disorders
ICD9	362.81	Retinal Hemorrhage
ICD9	362.82	Retinal Exudates and Deposits
ICD9	362.83	Retinal Edema
ICD9	362.84	Retinal Ischemia
ICD9	362.85	Retinal Nerve Fiber Bundle Defects
ICD9	362.89	Other Retinal Disorders
ICD9	362.9	Unspecified Retinal Disorder
ICD9	363	Chorioretinal Inflammations, Scars, and Other Disorders of Choroid
ICD9	363	Focal Chorioretinitis and Focal Retinochoroiditis
ICD9	363	Focal Chorioretinitis, Unspecified
ICD9	363.01	Focal Choroiditis and Chorioretinitis, Juxtapapillary
ICD9	363.03	Focal Choroiditis and Chorioretinitis of Other Posterior Pole
ICD9	363.04	Focal Choroiditis and Chorioretinitis, Peripheral
ICD9	363.05	Focal Retinitis and Retinochoroiditis, Juxtapapillary
ICD9	363.06	Focal Retinitis and Retinochoroiditis, Macular or Paramacular
ICD9	363.07	Focal Retinitis and Retinochoroiditis of Other Posterior Pole

ICD type	ICD code	Details
ICD9	363.08	Focal Retinitis and Retinochoroiditis, Peripheral
ICD9	363.1	Disseminated Chorioretinitis and Disseminated Retinochoroiditis
ICD9	363.1	Disseminated Chorioretinitis, Unspecified
ICD9	363.11	Disseminated Choroiditis and Chorioretinitis, Posterior Pole
ICD9	363.12	Disseminated Choroiditis and Chorioretinitis, Peripheral
ICD9	363.13	Disseminated Choroiditis and Chorioretinitis, Generalized
ICD9	363.14	Disseminated Retinitis and Retinochoroiditis, Metastatic
ICD9	363.15	Disseminated Retinitis and Retinochoroiditis, Pigment Epitheliopathy
ICD9	363.2	Other and Unspecified Forms of Chorioretinitis and Retinochoroiditis
ICD9	363.2	Chorioretinitis, Unspecified
ICD9	363.3	Chorioretinal Scars
ICD9	363.3	Chorioretinal Scar, Unspecified
ICD9	363.32	Other Macular Scars of Retina
ICD9	363.33	Other Scars of Posterior Pole of Retina
ICD9	363.34	Peripheral Scars of Retina
ICD9	363.35	Disseminated Scars of Retina
ICD9	363.4	Choroidal Degenerations
ICD9	363.4	Choroidal Degeneration, Unspecified
ICD9	363.5	Hereditary Choroidal Dystrophies
ICD9	363.5	Hereditary Choroidal Dystrophy or Atrophy, Unspecified
ICD9	363.54	Central Choroidal Atrophy, Total

ICD type	ICD code	Details
ICD9	363.6	Choroidal Hemorrhage and Rupture
ICD9	363.61	Choroidal Hemorrhage, Unspecified
ICD9	363.62	Expulsive Choroidal Hemorrhage
ICD9	363.63	Choroidal Rupture
ICD9	363.7	Choroidal Detachment
ICD9	363.7	Choroidal Detachment, Unspecified
ICD9	363.71	Serous Choroidal Detachment
ICD9	363.72	Hemorrhagic Choroidal Detachment
ICD9	365	Glaucoma
ICD9	365	Borderline Glaucoma [glaucoma Suspect]
ICD9	365.02	Anatomical Narrow Angle Borderline Glaucoma
ICD9	365.03	Steroid Responders Borderline Glaucoma
ICD9	365.06	Primary Angle Closure without Glaucoma Damage
ICD9	365.1	Open-Angle Glaucoma
ICD9	365.1	Open-Angle Glaucoma, Unspecified
ICD9	365.11	Primary Open Angle Glaucoma
ICD9	365.12	Low Tension Glaucoma
ICD9	365.13	Pigmentary Glaucoma
ICD9	365.14	Glaucoma of Childhood
ICD9	365.15	Residual Stage of Open Angle Glaucoma
ICD9	365.2	Primary Angle-Closure Glaucoma
ICD9	365.2	Primary Angle-Closure Glaucoma, Unspecified
ICD9	365.21	Intermittent Angle-Closure Glaucoma

ICD type	ICD code	Details
ICD9	365.22	Acute Angle-Closure Glaucoma
ICD9	365.23	Chronic Angle-Closure Glaucoma
ICD9	365.24	Residual Stage of Angle-Closure Glaucoma
ICD9	365.3	Corticosteroid-Induced Glaucoma
ICD9	365.31	Corticosteroid-Induced Glaucoma, Glaucomatous Stage
ICD9	365.32	Corticosteroid-Induced Glaucoma, Residual Stage
ICD9	365.4	Glaucoma Associated with Congenital Anomalies, Dystrophies, and Systemic Syndromes
ICD9	365.41	Glaucoma Associated with Chamber Angle Anomalies
ICD9	365.42	Glaucoma Associated with Anomalies of Iris
ICD9	365.43	Glaucoma Associated with Other Anterior Segment Anomalies
ICD9	365.44	Glaucoma Associated with Systemic Syndromes
ICD9	365.5	Glaucoma Associated with Disorders of the Lens
ICD9	365.51	Phacolytic Glaucoma
ICD9	365.52	Pseudoexfoliation Glaucoma
ICD9	365.59	Glaucoma Associated with Other Lens Disorders
ICD9	365.6	Glaucoma Associated with Other Ocular Disorders
ICD9	365.6	Glaucoma Associated with Unspecified Ocular Disorder
ICD9	365.61	Glaucoma Associated with Pupillary Block
ICD9	365.62	Glaucoma Associated with Ocular Inflammations
ICD9	365.63	Glaucoma Associated with Vascular Disorders
ICD9	365.64	Glaucoma Associated with Tumors or Cysts
ICD9	365.65	Glaucoma Associated with Ocular Trauma

ICD type	ICD code	Details
ICD9	365.7	Glaucoma Stage
ICD9	365.7	Glaucoma Stage, Unspecified
ICD9	365.71	Mild Stage Glaucoma
ICD9	365.72	Moderate Stage Glaucoma
ICD9	365.73	Severe Stage Glaucoma
ICD9	365.74	Indeterminate Stage Glaucoma
ICD9	365.8	Other Specified Forms of Glaucoma
ICD9	365.81	Hypersecretion Glaucoma
ICD9	365.82	Glaucoma with Increased Episcleral Venous Pressure
ICD9	365.89	Other Specified Glaucoma
ICD9	365.9	Unspecified Glaucoma
ICD9	368.34	Abnormal Retinal Correspondence
ICD9	377.03	Papilledema Associated with Retinal Disorder
ICD9	377.13	Optic Atrophy Associated with Retinal Dystrophies
ICD9	743.53	Chorioretinal Degeneration, Congenital
ICD9	743.56	Other Retinal Changes, Congenital
ICD9	794.11	Nonspecific Abnormal Retinal Function Studies
ICD9	V19.11	Family History of Glaucoma
ICD9	V80.1	Screening for Glaucoma
ICD10-CM	H30	Chorioretinal inflammation
ICD10-CM	H31.0	Chorioretinal scars
ICD10-CM	H31.1	Choroidal degeneration
ICD10-CM	H31.101	Choroidal degeneration, unspecified, right eye

ICD type	ICD code	Details
ICD10-CM	H31.102	Choroidal degeneration, unspecified, left eye
ICD10-CM	H31.103	Choroidal degeneration, unspecified, bilateral
ICD10-CM	H31.109	Choroidal degeneration, unspecified, unspecified eye
ICD10-CM	H31.22	Choroidal dystrophy (central areolar) (generalized) (peripapillary)
ICD10-CM	H31.3	Choroidal hemorrhage and rupture
ICD10-CM	H31.32	Choroidal rupture
ICD10-CM	H31.321	Choroidal rupture, right eye
ICD10-CM	H31.322	Choroidal rupture, left eye
ICD10-CM	H31.323	Choroidal rupture, bilateral
ICD10-CM	H31.329	Choroidal rupture, unspecified eye
ICD10-CM	H31.4	Choroidal detachment
ICD10-CM	H32	Chorioretinal disorders in diseases classified elsewhere
ICD10-CM	H33	Retinal detachments and breaks
ICD10-CM	H33.0	Retinal detachment with retinal break
ICD10-CM	H33.01	Retinal detachment with single break
ICD10-CM	H33.011	Retinal detachment with single break, right eye
ICD10-CM	H33.012	Retinal detachment with single break, left eye
ICD10-CM	H33.013	Retinal detachment with single break, bilateral
ICD10-CM	H33.019	Retinal detachment with single break, unspecified eye
ICD10-CM	H33.02	Retinal detachment with multiple breaks
ICD10-CM	H33.021	Retinal detachment with multiple breaks, right eye
ICD10-CM	H33.022	Retinal detachment with multiple breaks, left eye

ICD type	ICD code	Details
ICD10-CM	H33.023	Retinal detachment with multiple breaks, bilateral
ICD10-CM	H33.029	Retinal detachment with multiple breaks, unspecified eye
ICD10-CM	H33.03	Retinal detachment with giant retinal tear
ICD10-CM	H33.031	Retinal detachment with giant retinal tear, right eye
ICD10-CM	H33.032	Retinal detachment with giant retinal tear, left eye
ICD10-CM	H33.033	Retinal detachment with giant retinal tear, bilateral
ICD10-CM	H33.039	Retinal detachment with giant retinal tear, unspecified eye
ICD10-CM	H33.04	Retinal detachment with retinal dialysis
ICD10-CM	H33.041	Retinal detachment with retinal dialysis, right eye
ICD10-CM	H33.042	Retinal detachment with retinal dialysis, left eye
ICD10-CM	H33.043	Retinal detachment with retinal dialysis, bilateral
ICD10-CM	H33.049	Retinal detachment with retinal dialysis, unspecified eye
ICD10-CM	H33.3	Retinal breaks without detachment
ICD10-CM	H34	Retinal vascular occlusions
ICD10-CM	H34.23	Retinal artery branch occlusion
ICD10-CM	H34.231	Retinal artery branch occlusion, right eye
ICD10-CM	H34.232	Retinal artery branch occlusion, left eye
ICD10-CM	H34.233	Retinal artery branch occlusion, bilateral
ICD10-CM	H34.239	Retinal artery branch occlusion, unspecified eye
ICD10-CM	H35.04	Retinal micro-aneurysms, unspecified
ICD10-CM	H35.041	Retinal micro-aneurysms, unspecified, right eye
ICD10-CM	H35.042	Retinal micro-aneurysms, unspecified, left eye
ICD10-CM	H35.043	Retinal micro-aneurysms, unspecified, bilateral

ICD type	ICD code	Details
ICD10-CM	H35.049	Retinal micro-aneurysms, unspecified, unspecified eye
ICD10-CM	H35.05	Retinal neovascularization, unspecified
ICD10-CM	H35.051	Retinal neovascularization, unspecified, right eye
ICD10-CM	H35.052	Retinal neovascularization, unspecified, left eye
ICD10-CM	H35.053	Retinal neovascularization, unspecified, bilateral
ICD10-CM	H35.059	Retinal neovascularization, unspecified, unspecified eye
ICD10-CM	H35.06	Retinal vasculitis
ICD10-CM	H35.061	Retinal vasculitis, right eye
ICD10-CM	H35.062	Retinal vasculitis, left eye
ICD10-CM	H35.063	Retinal vasculitis, bilateral
ICD10-CM	H35.069	Retinal vasculitis, unspecified eye
ICD10-CM	H35.07	Retinal telangiectasis
ICD10-CM	H35.071	Retinal telangiectasis, right eye
ICD10-CM	H35.072	Retinal telangiectasis, left eye
ICD10-CM	H35.073	Retinal telangiectasis, bilateral
ICD10-CM	H35.079	Retinal telangiectasis, unspecified eye
ICD10-CM	H35.6	Retinal hemorrhage
ICD10-CM	H35.60	Retinal hemorrhage, unspecified eye
ICD10-CM	H35.61	Retinal hemorrhage, right eye
ICD10-CM	H35.62	Retinal hemorrhage, left eye
ICD10-CM	H35.63	Retinal hemorrhage, bilateral
ICD10-CM	H35.81	Retinal edema
ICD10-CM	H35.82	Retinal ischemia

ICD type	ICD code	Details
ICD10-CM	H36	Retinal disorders in diseases classified elsewhere
ICD10-CM	H40	Glaucoma
ICD10-CM	H40.0	Glaucoma suspect
ICD10-CM	H40.3	Glaucoma secondary to eye trauma
ICD10-CM	H40.30	Glaucoma secondary to eye trauma, unspecified eye
ICD10-CM	H40.30X0	Glaucoma secondary to eye trauma, unspecified eye, stage unspecified
ICD10-CM	H40.30X1	Glaucoma secondary to eye trauma, unspecified eye, mild stage
ICD10-CM	H40.30X2	Glaucoma secondary to eye trauma, unspecified eye, moderate stage
ICD10-CM	H40.30X3	Glaucoma secondary to eye trauma, unspecified eye, severe stage
ICD10-CM	H40.30X4	Glaucoma secondary to eye trauma, unspecified eye, indeterminate stage
ICD10-CM	H40.31	Glaucoma secondary to eye trauma, right eye
ICD10-CM	H40.31X0	Glaucoma secondary to eye trauma, right eye, stage unspecified
ICD10-CM	H40.31X1	Glaucoma secondary to eye trauma, right eye, mild stage
ICD10-CM	H40.31X2	Glaucoma secondary to eye trauma, right eye, moderate stage
ICD10-CM	H40.31X3	Glaucoma secondary to eye trauma, right eye, severe stage
ICD10-CM	H40.31X4	Glaucoma secondary to eye trauma, right eye, indeterminate stage
ICD10-CM	H40.32	Glaucoma secondary to eye trauma, left eye
ICD10-CM	H40.32X0	Glaucoma secondary to eye trauma, left eye, stage unspecified

ICD type	ICD code	Details
ICD10-CM	H40.32X1	Glaucoma secondary to eye trauma, left eye, mild stage
ICD10-CM	H40.32X2	Glaucoma secondary to eye trauma, left eye, moderate stage
ICD10-CM	H40.32X3	Glaucoma secondary to eye trauma, left eye, severe stage
ICD10-CM	H40.32X4	Glaucoma secondary to eye trauma, left eye, indeterminate stage
ICD10-CM	H40.33	Glaucoma secondary to eye trauma, bilateral
ICD10-CM	H40.33X0	Glaucoma secondary to eye trauma, bilateral, stage unspecified
ICD10-CM	H40.33X1	Glaucoma secondary to eye trauma, bilateral, mild stage
ICD10-CM	H40.33X2	Glaucoma secondary to eye trauma, bilateral, moderate stage
ICD10-CM	H40.33X3	Glaucoma secondary to eye trauma, bilateral, severe stage
ICD10-CM	H40.33X4	Glaucoma secondary to eye trauma, bilateral, indeterminate stage
ICD10-CM	H40.4	Glaucoma secondary to eye inflammation
ICD10-CM	H40.40	Glaucoma secondary to eye inflammation, unspecified eye
ICD10-CM	H40.40X0	Glaucoma secondary to eye inflammation, unspecified eye, stage unspecified
ICD10-CM	H40.40X1	Glaucoma secondary to eye inflammation, unspecified eye, mild stage
ICD10-CM	H40.40X2	Glaucoma secondary to eye inflammation, unspecified eye, moderate stage
ICD10-CM	H40.40X3	Glaucoma secondary to eye inflammation, unspecified eye, severe stage
ICD10-CM	H40.40X4	Glaucoma secondary to eye inflammation, unspecified eye, indeterminate stage

ICD type	ICD code	Details
ICD10-CM	H40.41	Glaucoma secondary to eye inflammation, right eye
ICD10-CM	H40.41X0	Glaucoma secondary to eye inflammation, right eye, stage unspecified
ICD10-CM	H40.41X1	Glaucoma secondary to eye inflammation, right eye, mild stage
ICD10-CM	H40.41X2	Glaucoma secondary to eye inflammation, right eye, moderate stage
ICD10-CM	H40.41X3	Glaucoma secondary to eye inflammation, right eye, severe stage
ICD10-CM	H40.41X4	Glaucoma secondary to eye inflammation, right eye, indeterminate stage
ICD10-CM	H40.42	Glaucoma secondary to eye inflammation, left eye
ICD10-CM	H40.42X0	Glaucoma secondary to eye inflammation, left eye, stage unspecified
ICD10-CM	H40.42X1	Glaucoma secondary to eye inflammation, left eye, mild stage
ICD10-CM	H40.42X2	Glaucoma secondary to eye inflammation, left eye, moderate stage
ICD10-CM	H40.42X3	Glaucoma secondary to eye inflammation, left eye, severe stage
ICD10-CM	H40.42X4	Glaucoma secondary to eye inflammation, left eye, indeterminate stage
ICD10-CM	H40.43	Glaucoma secondary to eye inflammation, bilateral
ICD10-CM	H40.43X0	Glaucoma secondary to eye inflammation, bilateral, stage unspecified
ICD10-CM	H40.43X1	Glaucoma secondary to eye inflammation, bilateral, mild stage

ICD type	ICD code	Details
ICD10-CM	H40.43X2	Glaucoma secondary to eye inflammation, bilateral, moderate stage
ICD10-CM	H40.43X3	Glaucoma secondary to eye inflammation, bilateral, severe stage
ICD10-CM	H40.43X4	Glaucoma secondary to eye inflammation, bilateral, indeterminate stage
ICD10-CM	H40.5	Glaucoma secondary to other eye disorders
ICD10-CM	H40.50	Glaucoma secondary to other eye disorders, unspecified eye
ICD10-CM	H40.50X0	Glaucoma secondary to other eye disorders, unspecified eye, stage unspecified
ICD10-CM	H40.50X1	Glaucoma secondary to other eye disorders, unspecified eye, mild stage
ICD10-CM	H40.50X2	Glaucoma secondary to other eye disorders, unspecified eye, moderate stage
ICD10-CM	H40.50X3	Glaucoma secondary to other eye disorders, unspecified eye, severe stage
ICD10-CM	H40.50X4	Glaucoma secondary to other eye disorders, unspecified eye, indeterminate stage
ICD10-CM	H40.51	Glaucoma secondary to other eye disorders, right eye
ICD10-CM	H40.51X0	Glaucoma secondary to other eye disorders, right eye, stage unspecified
ICD10-CM	H40.51X1	Glaucoma secondary to other eye disorders, right eye, mild stage
ICD10-CM	H40.51X2	Glaucoma secondary to other eye disorders, right eye, moderate stage

ICD type	ICD code	Details
ICD10-CM	H40.51X3	Glaucoma secondary to other eye disorders, right eye, severe stage
ICD10-CM	H40.51X4	Glaucoma secondary to other eye disorders, right eye, indeterminate stage
ICD10-CM	H40.52	Glaucoma secondary to other eye disorders, left eye
ICD10-CM	H40.52X0	Glaucoma secondary to other eye disorders, left eye, stage unspecified
ICD10-CM	H40.52X1	Glaucoma secondary to other eye disorders, left eye, mild stage
ICD10-CM	H40.52X2	Glaucoma secondary to other eye disorders, left eye, moderate stage
ICD10-CM	H40.52X3	Glaucoma secondary to other eye disorders, left eye, severe stage
ICD10-CM	H40.52X4	Glaucoma secondary to other eye disorders, left eye, indeterminate stage
ICD10-CM	H40.53	Glaucoma secondary to other eye disorders, bilateral
ICD10-CM	H40.53X0	Glaucoma secondary to other eye disorders, bilateral, stage unspecified
ICD10-CM	H40.53X1	Glaucoma secondary to other eye disorders, bilateral, mild stage
ICD10-CM	H40.53X2	Glaucoma secondary to other eye disorders, bilateral, moderate stage
ICD10-CM	H40.53X3	Glaucoma secondary to other eye disorders, bilateral, severe stage
ICD10-CM	H40.53X4	Glaucoma secondary to other eye disorders, bilateral, indeterminate stage

ICD type	ICD code	Details
ICD10-CM	H40.6	Glaucoma secondary to drugs
ICD10-CM	H40.60	Glaucoma secondary to drugs, unspecified eye
ICD10-CM	H40.60X0	Glaucoma secondary to drugs, unspecified eye, stage unspecified
ICD10-CM	H40.60X1	Glaucoma secondary to drugs, unspecified eye, mild stage
ICD10-CM	H40.60X2	Glaucoma secondary to drugs, unspecified eye, moderate stage
ICD10-CM	H40.60X3	Glaucoma secondary to drugs, unspecified eye, severe stage
ICD10-CM	H40.60X4	Glaucoma secondary to drugs, unspecified eye, indeterminate stage
ICD10-CM	H40.61	Glaucoma secondary to drugs, right eye
ICD10-CM	H40.61X0	Glaucoma secondary to drugs, right eye, stage unspecified
ICD10-CM	H40.61X1	Glaucoma secondary to drugs, right eye, mild stage
ICD10-CM	H40.61X2	Glaucoma secondary to drugs, right eye, moderate stage
ICD10-CM	H40.61X3	Glaucoma secondary to drugs, right eye, severe stage
ICD10-CM	H40.61X4	Glaucoma secondary to drugs, right eye, indeterminate stage
ICD10-CM	H40.62	Glaucoma secondary to drugs, left eye
ICD10-CM	H40.62X0	Glaucoma secondary to drugs, left eye, stage unspecified
ICD10-CM	H40.62X1	Glaucoma secondary to drugs, left eye, mild stage
ICD10-CM	H40.62X2	Glaucoma secondary to drugs, left eye, moderate stage
ICD10-CM	H40.62X3	Glaucoma secondary to drugs, left eye, severe stage
ICD10-CM	H40.62X4	Glaucoma secondary to drugs, left eye, indeterminate stage
ICD10-CM	H40.63	Glaucoma secondary to drugs, bilateral
ICD10-CM	H40.63X0	Glaucoma secondary to drugs, bilateral, stage unspecified

ICD type	ICD code	Details
ICD10-CM	H40.63X1	Glaucoma secondary to drugs, bilateral, mild stage
ICD10-CM	H40.63X2	Glaucoma secondary to drugs, bilateral, moderate stage
ICD10-CM	H40.63X3	Glaucoma secondary to drugs, bilateral, severe stage
ICD10-CM	H40.63X4	Glaucoma secondary to drugs, bilateral, indeterminate stage
ICD10-CM	H40.81	Glaucoma with increased episcleral venous pressure
ICD10-CM	H40.811	Glaucoma with increased episcleral venous pressure, right eye
ICD10-CM	H40.812	Glaucoma with increased episcleral venous pressure, left eye
ICD10-CM	H40.813	Glaucoma with increased episcleral venous pressure, bilateral
ICD10-CM	H40.819	Glaucoma with increased episcleral venous pressure, unspecified eye
ICD10-CM	H42	Glaucoma in diseases classified elsewhere
ICD10-CM	H59.81	Chorioretinal scars after surgery for detachment
ICD10-CM	H59.811	Chorioretinal scars after surgery for detachment, right eye
ICD10-CM	H59.812	Chorioretinal scars after surgery for detachment, left eye
ICD10-CM	H59.813	Chorioretinal scars after surgery for detachment, bilateral
ICD10-CM	H59.819	Chorioretinal scars after surgery for detachment, unspecified eye

IV. Medication exposure

Thirty-day exposure, by medication group, prior to the index date

**Medication group**	**Mean (±SD)** or No. (%) of filled prescriptions		**p-value**
	**Cases**	**Controls**	
**Quinolones**	**0.044 (±0.28)**	**0.037 (±0.26)**	**<0.001**
0	748 (96.9%)	3767 (97.6%)	0.2791
1-3	23 (3.0%)	92 (2.4%)	
4-6	1 (0.1%)	1 (0.0%)	
**Other antibiotics**	**0.22 (±0.92)**	**0.11 (±0.52)**	**<0.001**
0	706 (91.5%)	3643 (94.4%)	<.0013
1-3	53 (6.9%)	194 (5.0%)	
4-6	11 (1.4%)	22 (0.6%)	
7+	2 (0.3%)	1 (0.0%)	
**Other medications**	**4.90 (±14.43)**	**3.04 (±10.02)**	**<0.001**
0	446 (57.8%)	2700 (70.0%)	<.0001
1-3	207 (26.8%)	774 (20.1%)	

**Medication group**	**Mean (±SD)** or No. (%) of filled prescriptions		**p-value**
	**Cases**	**Controls**	
4-6	14 (1.8%)	63 (1.6%)	
7-10	10 (1.3%)	36 (0.9%)	
11+	95 (12.3%)	287 (7.4%)	

Thirty-day medication exposure by the individual quinolones

Quinolone antibiotic	No. (%) of filled prescriptions		p-value
	Cases	Controls	
**Ciprofloxacin**	12 (1.6%)	37 (1.0%)	0.14
**Levofloxacin**	11 (1.4%)	56 (1.5%)	0.96
**Moxifloxacin**	1 (0.1%)	3 (0.1%)	0.66

V. Odds ratio and 95% CL for quinolones and other medication groups, and risk of retinal detachment

Population and medication group	Base[1] OR (95% CI)	p-value	Minimally adjusted[2] maOR (95% CI)	p-value	Maximally adjusted[3] aOR (95% CI)	p-value
**Entire population**						
Quinolones	1.27 (0.80-2.01)	0.3055	0.84 (0.51-1.38)	0.4907	0.75 (0.43-1.32)	0.3184
Non-quinolone antibiotics	1.54 (1.15-2.06)	0.0036	1.18 (0.85-1.64)	0.3117	0.86 (0.58-1.26)	0.4259
**Comorbidity level**						
CMI:0-1						
Quinolones	1.40 (0.25-7.79)	0.6984	0.72 (0.12-4.39)	0.7212	0.70 (0.05-9.74)	0.7914
Non-quinolone antibiotics	2.66 (1.00-7.08)	0.0503	2.09 (0.72-6.05)	0.1753	1.12 (0.28-4.44)	0.8777
CMI:2-4						
Quinolones	0.46 (0.09-2.29)	0.3420	0.48 (0.09-2.50)	0.3842	0.35 (0.05-2.42)	0.2872

[1] Base model: age, sex, race variables, and the tested medication group
[2] Minimally adjusted model: base model, and the other medication groups
[3] Maximally adjusted model: minimally adjusted, and complicated diabetes mellitus, alcohol abuse and socioeconomic status (census division, hospital (urban/rural) and insurance)

Population and medication group	Base[1] OR (95% CI)	p-value	Minimally adjusted[2] maOR (95% CI)	p-value	Maximally adjusted[3] aOR (95% CI)	p-value
Non-quinolone antibiotics	0.62 (0.22-1.79)	0.3782	0.63 (0.21-1.90)	0.4149	0.49 (0.12-1.92)	0.3053
CMI:5+						
Quinolones	0.87 (0.45-1.66)	0.6624	0.60 (0.29-1.24)	0.1641	0.49 (0.21-1.17)	0.1094
Non-quinolone antibiotics	1.46 (0.91-2.33)	0.1173	1.19 (0.67-2.11)	0.5635	1.15 (0.55-2.38)	0.7155
**Gender**						
Women						
Quinolones	1.22 (0.60-2.46)	0.5860	0.90 (0.41-1.95)	0.7894	0.89 (0.36-2.23)	0.8042
Non-quinolone antibiotics	1.23 (0.78-1.94)	0.3812	0.90 (0.54-1.50)	0.6759	0.62 (0.34-1.15)	0.1307
Men						
Quinolones	1.44 (0.79-2.64)	0.2367	0.89 (0.46-1.73)	0.7309	0.72 (0.34-1.51)	0.3826
Non-quinolone antibiotics	1.84 (1.26-2.69)	0.0018	1.43 (0.93-2.19)	0.1059	1.10 (0.66-1.82)	0.7198
**Race Class**	Race Class					

**Population and medication group**	**Base[1] OR (95% CI)**	**p-value**	**Minimally adjusted[2] maOR (95% CI)**	**p-value**	**Maximally adjusted[3] aOR (95% CI)**	**p-value**
Caucasians	Caucasians					
Quinolones	1.12 (0.67-1.89)	0.6638	0.75 (0.42-1.32)	0.3145	0.67 (0.36-1.24)	0.2013
Non-quinolone antibiotics	1.55 (1.13-2.14)	0.0071	1.30 (0.91-1.87)	0.1558	1.06 (0.70-1.59)	0.8005
African Americans						
Quinolones	3.57 (1.13-11.25)	0.0297	2.53 (0.69-9.28)	0.1609	2.88 (0.43-19.33)	0.2758
Non-quinolone antibiotics	1.66 (0.74-3.76)	0.2213	0.77 (0.30-1.98)	0.5916	0.13 (0.03-0.58)	0.0076
Others						
Quinolones	0.71 (0.08-6.18)	0.7565	0.45 (0.03-6.19)	0.5481	0.001 (<0.001-0.147)	0.0062
Non-quinolone antibiotics	1.15 (0.31-4.22)	0.8369	1.29 (0.25-6.54)	0.7588	13.16 (0.75-230.14)	0.0776
**Age Stratum**						
0-55						
Quinolones	4.01 (1.78-9.05)	0.0008	2.13 (0.88-5.14)	0.0924	0.87 (0.26-2.93)	0.8156

Population and medication group	Base[1] OR (95% CI)	p-value	Minimally adjusted[2] maOR (95% CI)	p-value	Maximally adjusted[3] aOR (95% CI)	p-value
Non-quinolone antibiotics	2.51 (1.50-4.19)	0.0004	1.50 (0.85-2.66)	<.0001	0.76 (0.32-1.79)	0.5337
56-70						
Quinolones	1.03 (0.46-2.35)	0.9360	0.92 (0.37-2.29)	0.8516	0.90 (0.34-2.41)	0.8404
Non-quinolone antibiotics	1.07 (0.62-1.83)	0.8179	0.85 (0.46-1.59)	0.6112	0.68 (0.34-1.36)	0.2733
71+						
Quinolones	0.66 (0.27-1.57)	1.5680	0.41 (0.16-1.05)	0.0622	0.42 (0.16-1.15)	0.0916
Non-quinolone antibiotics	1.47 (0.91-2.37)	0.1172	1.34 (0.78-2.29)	0.2852	1.13 (0.61-2.08)	0.7021

VI. Odds ratio and 95% CL for individual quinolones and risk of retinal detachment

Population and individual quinolone	Base OR (95% CI)	p-value	Minimally adjusted maOR (95% CI)	p-value	Maximally adjusted aOR (95% CI)	p-value
**Entire Population**						
Ciprofloxacin	1.60 (0.83-3.07)	0.1588	1.07 (0.54-2.14)	0.8399	0.87 (0.39-1.97)	0.7415
Levofloxacin	0.94 (0.48-1.81)	0.8411	0.62 (0.32-1.24)	0.1774	0.61 (0.29-1.30)	0.1984
Moxifloxacin	1.67 (0.17-16.02)	0.6582	1.25 (0.13-12.06)	0.8474	1.07 (0.10-11.08)	0.9535
**Comorbidity level**						
CMI:0-1						
Ciprofloxacin	2.78 (0.38-20.39)	0.3157	1.45 (0.18-11.48)	0.7233	1.00 (0.05-19.93)	0.9987
Levofloxacin	<0.001 (<0.001->999.999)	0.9826	<0.001 (<0.001->999.999)	0.9825	<0.001 (<0.001->999.999)	0.9905
Moxifloxacin	N/A	N/A	N/A	N/A	N/A	N/A
CMI:2-4						
Ciprofloxacin	0.40 (0.04-3.75)	0.4239	0.41 (0.04-3.88)	0.4344	0.15 (0.01-2.05)	0.1546
Levofloxacin	0.54 (0.05-5.47)	0.5981	0.59 (0.05-6.43)	0.6657	1.07 (0.08-13.78)	0.9577
Moxifloxacin	N/A	N/A	N/A	N/A	N/A	N/A
CMI:5+						

Population and individual quinolone	Base OR (95% CI)	p-value	Minimally adjusted maOR (95% CI)	p-value	Maximally adjusted aOR (95% CI)	p-value
Ciprofloxacin	1.24 (0.44-3.43)	0.6858	0.83 (0.27-2.52)	0.7355	0.74 (0.19-2.91)	0.6606
Levofloxacin	0.70 (0.29-1.74)	0.4455	0.49 (0.19-1.27)	0.1397	0.46 (0.16-1.39)	0.1691
Moxifloxacin	0.65 (0.07-6.60)	0.7190	0.57 (0.06-5.66)	0.6335	0.14 (0.01-3.59)	0.2369
**Gender**						
Women						
Ciprofloxacin	1.65 (0.60-4.56)	0.3304	1.25 (0.43-3.71)	0.6820	1.34 (0.34-5.22)	0.6726
Levofloxacin	0.90 (0.34-2.39)	0.8358	0.68 (0.25-1.86)	0.4507	0.68 (0.22-2.15)	0.5150
Moxifloxacin	N/A	N/A	N/A	N/A	N/A	N/A
Men						
Ciprofloxacin	1.84 (0.77-4.39)	0.1676	1.18 (0.47-2.93)	0.7281	0.86 (0.30-2.48)	0.7855
Levofloxacin	1.00 (0.41-2.44)	0.9963	0.61 (0.24-1.56)	0.3027	0.52 (0.18-1.48)	0.2176
Moxifloxacin	1.67 (0.17-16.02)	0.6582	1.22 (0.13-11.83)	0.8657	1.12 (0.11-11.46)	0.9211
**Race Class**						
Caucasians						
Ciprofloxacin	1.38 (0.63-3.02)	0.4225	0.92 (0.41-2.10)	0.8492	0.80 (0.32-2.02)	0.6364

Population and individual quinolone	Base OR (95% CI)	p-value	Minimally adjusted maOR (95% CI)	p-value	Maximally adjusted aOR (95% CI)	p-value
Levofloxacin	0.96 (0.48-1.92)	0.9156	0.65 (0.32-1.34)	0.2441	0.60 (0.27-1.33)	0.2117
Moxifloxacin	<0.001 (<0.001->999.999)	0.9786	<0.001 (<0.001->999.999)	0.9779	<0.001 (<0.001->999.999)	0.9796
African Americans						
Ciprofloxacin	3.69 (0.79-17.17)	0.0960	3.07 (0.56-16.75)	0.1953	2.09 (0.11-41.09)	0.6268
Levofloxacin	1.12 (0.10-12.48)	0.9281	0.72 (0.07-7.93)	0.7883	1.85 (0.08-42.88)	0.7006
Moxifloxacin	5.00 (0.31-79.94)	0.2551	3.23 (0.20-51.95)	0.4081	0.84 (0.04-20.04)	0.9139
Others						
Ciprofloxacin	1.15 (0.12-10.97)	0.9030	0.72 (0.05-10.27)	0.8070	#VALUE!	0.0437
Levofloxacin	<0.001 (<0.001->999.999)	0.9904	<0.001 (<0.001->999.999)	0.9901	<0.001 (<0.001->999.999)	0.9972
Moxifloxacin	N/A	N/A	N/A	N/A	N/A	N/A
**Age Stratum**						
0-55						
Ciprofloxacin	5.91 (1.76-19.84)	0.0040	3.29 (0.93-11.69)	0.0657	0.83 (0.15-4.52)	0.8282
Levofloxacin	2.34 (0.73-7.47)	0.1514	1.28 (0.39-4.22)	0.6827	0.93 (0.18-4.95)	0.9343

Population and individual quinolone	Base OR (95% CI)	p-value	Minimally adjusted maOR (95% CI)	p-value	Maximally adjusted aOR (95% CI)	p-value
Moxifloxacin	<0.001 (<0.001->999.999)	0.9938	<0.001 (<0.001->999.999)	0.9938	<0.001 (<0.001->999.999)	0.9944
56-70						
Ciprofloxacin	0.84 (0.19-3.75)	0.8163	0.79 (0.17-3.78)	0.7660	0.84 (0.15-4.72)	0.8418
Levofloxacin	0.92 (0.31-2.69)	0.8765	0.78 (0.25-2.42)	0.6640	0.71 (0.21-2.38)	0.5827
Moxifloxacin	5.00 (0.31-79.94)	0.2551	4.50 (0.28-73.18)	0.2904	5.36 (0.30-97.21)	0.2558
71+						
Ciprofloxacin	1.08 (0.37-3.21)	0.8865	0.67 (0.22-2.09)	0.4910	0.64 (0.18-2.26)	0.4891
Levofloxacin	0.36 (0.08-1.60)	0.1791	0.24 (0.05-1.08)	0.0622	0.27 (0.06-1.29)	0.1009
Moxifloxacin	<0.001 (<0.001->999.999)	0.9906	<0.001 (<0.001->999.999)	0.9907	<0.001 (<0.001->999.999)	0.9907

VII. Odds ratios and 95% CI for complicated diabetes mellitus and alcohol abuse, and risk of retinal detachment

	Diabetes mellitus, complicated	p-value	Alcohol abuse	p-value
**Entire population**	2.19 (1.68-2.86)	<.0001	1.03 (0.67-1.58)	0.8872
**Comorbidity score level**				
CMI: 0-1	14.08 (1.40-141.93)	0.0249	0.69 (0.01-90.55)	0.8825
CMI: 2-4	0.46 (0.11-1.98)	0.0654	0.55 (0.14-2.13)	0.2999
CMI: 5+	1.94 (1.19-3.18)	0.0083	0.82 (0.38-1.77)	0.6098
**Sex**				
Women	2.69 (1.82-4.00)	<.0001	1.56 (0.67-3.59)	0.2999
Men	1.82 (1.26-2.63)	0.0014	0.99 (0.59-1.64)	0.9644
**Race**				
Caucasians	1.69 (1.24-2.30)	0.0008	1.23 (0.76-1.97)	0.3986
African Americans	5.85 (2.70-12.65)	<.0001	0.90 (0.26-3.16)	0.8728
Others	21.84 (4.25-112.28)	0.0002	<0.001 (<0.001->999.999)	0.9932

	Diabetes mellitus, complicated	p-value	Alcohol abuse	p-value
**Age (tertiles)**				
0-55	6.18 (3.65-10.46)	<.0001	1.02 (0.46-2.27)	0.9633
56-70	1.24 (0.80-1.92)	0.3453	0.99 (0.52-1.89)	0.9750
71+	1.60 (0.94-2.71)	0.0830	1.79 (0.74-4.33)	0.1978

VIII. Results from recent studies on quinolone antibiotics and risk of retinal detachment

**Study (Country)**	**Database**	**Design (Type)**	**Duration**	**Population**	**Age (mean ±SD) years Sex (%)**	**RD risk (formulation)**	**Agent Risk ratio (95% CI)**
Gatti 2020 [1]		Umbrella review (3 systematic reviews)				No association (systemic)	
Yu 2019 [2]		Systematic review (8 studies)				No association with current use (systemic)	Current use (1-30 days) OR: 1.25 (1.01–1.53) Past use (31-365 days) OR: 1.27 (1.09–1.47)
Choi 2018 [3] (South Korea)	Korean National Health Insurance Service National Sample Cohort (KNHIS-NSC)	Nested case-control (population-based)	2002 - 2013	Cases: 1,151 Controls: 11,470	NR Men: 56.4%	No association (oral)	aOR: 1.00 (0.81-1.24), P = 0.99
Alves 2016 [4]		Systematic review (10 studies)				No association (systemic)	RR: 1.47 (0.95–2.27) I_2 = 92.8%, p = 0.09

Study (Country)	Database	Design (Type)	Duration	Population	Age (mean ±SD) years Sex (%)	RD risk (formulation)	Agent Risk ratio (95% CI)
Raguideau 2016 [5] (France)	French National Interscheme Health Insurance Information System database (SNIIRAM)	Case-crossover (population-based)	1 Jul 2010 - 31 Dec 201	27,540 adults with RD	61.5 ± 13.6 Men: 57%	Increased risk for current but not recent use (oral)	Current use (10 days): aOR: 1.46 (1.15-1.87) Recent use (11-30 days): aOR: 0.94 (0.78-1.14)
Chui 2015 [6]		Systematic review (7 studies)				No association (systemic)	Current use: Overall absolute risk: 4.85 cases / 1 million prescriptions (95% CI: 0.78–8.91)
Eftekhari 2014 [7] (UK)	The Health Improvement Network (THIN) database	Cohort study (population-based)	Jun 1994 – Jan 2012	Patients who were prescribed fluoroquinolones or beta-lactam antibiotics	Quinolones: 55.1 years (1-109) Beta-lactams: 40 years (1-111) Men: 44%	No association (oral)	≥ 30-days: HR= 0.78 (0.11–5.71) 31-90 days: HR= 1.25 (0.51–3.08) 91-365 days: HR= 1.35 (0.89–2.06).
Fife 2014 [8]	MarketScan Commercial	Case-control study	Jan 2000 – Jan 2012			No association	Current use:

Study (Country)	Database	Design (Type)	Duration	Population	Age (mean ±SD) years<br>Sex (%)	RD risk (formulation)	Agent<br>Risk ratio (95% CI)
(USA)	Claims and Encounters database (CCAE)					(oral)	OR: 1.33 (0.99-1.80)<br>Recent use (7 days)<br>OR: 1.19 (0.81-1.76)
	Optum ClinFormatics database	Case-control study	Sep 2005 – Mar 2012			No association<br>(oral)	Current use:<br>OR: 0.93 (0.48-1.81)<br>Recent use (7 days)<br>OR: 0.74 (0.33-1.63)
	MarketScan Commercial Claims and Encounters database (CCAE)	Self-controlled case series	Jan 2000 – Jan 2012			No association<br>(oral)	30-day risk period:<br>IRR 1.13 (0.99-1.29)
	Optum ClinFormatics database	Self-controlled case series	Jan 2005 – Mar 2012			No association<br>(oral)	30-day risk period:<br>IRR: 0.85 (0.66-1.09)
Kapoor 2014 [9]<br>(USA)	Medical record linkage system Rochester	Cohort study (population-based)	1 Jan 2003 – 30 Jun 2011	Adult residents of Olmsted County, Minnesota who were	50.6 ± 19.6<br>Men: 39%	No association<br>(oral)	Recent use (30 days) *<br>RR: 1.82 (0.11-29.03)<br>Past use (90 days) *

Study (Country)	Database	Design (Type)	Duration	Population	Age (mean ±SD) years<br>Sex (%)	RD risk (formulation)	Agent<br>Risk ratio (95% CI)
	Epidemiology Project (REP)			prescribed oral fluoroquinolones			RR: 1.08 (0.26-4.56)
Kuo 2014 [10]<br>(Taiwan)	Taiwan National Health Insurance Research Database	Cohort study (population-based)	1998 – 2010	Adults (aged >18 years) who were prescribed >3 consecutive doses of an oral fluoroquinolone or amoxicillin) on study entry.<br>Fluoroquinolones cohort: 178,179<br>Amoxicillin cohort: 178,179	47.2 ± 18.3<br>Men: 40.8%	Increased risk<br>(oral)	Quinolones:<br>aHR: 2.07 (1.45–2.96).<br>Ciprofloxacin:<br>aHR: 10.68 (3.28–34.82)<br>Levofloxacin:<br>aHR: 2.41 (0.76–7.68)<br>Norfloxacin:<br>aHR: 2.00 (1.06–3.79)<br>Ofloxacin<br>aHR: 1.17 (0.59–2.31)
Pasternak 2013 [11]<br>(Denmark)	The Central Person Register of Denmark	Cohort study (population-based)	1 Jan 1997 – 31 Dec 2011	Adults (aged >18 years) with no prior RD<br>748,792 episodes of fluoroquinolone use (ciprofloxacin: 88%)	57.7 ± 19.9<br>Men: 38%	No association<br>(oral)	(10 days):<br>aRR: 1.29 (0.53 – 3.13)<br>(11-30 days):<br>aRR: 0.97 (0.46 – 2.05)

**Study (Country)**	**Database**	**Design (Type)**	**Duration**	**Population**	**Age (mean ±SD) years Sex (%)**	**RD risk (formulation)**	**Agent Risk ratio (95% CI)**
Etminan 2012 [12] (Canada)	British Columbia Linked Health Database	Nested case-control	1 Jan 2000 – 31 Dec 2007	Cases: 4,384<br>Controls: 43,840	61.1 ± 16.6<br>Men: 58.2%	Increased risk (oral)	Current use (overlaps index date):<br>aRR: 4.50 (3.56-5.70)<br>Recent use (1-7 days):<br>aRR: 0.92 (0.45-1.87)<br>Past use (8-365 days):<br>aRR: 1.03 (0.89-1.19]

***aHR:** adjusted hazard ratio**; aOR:** adjusted odds ratio, **aRR:** adjusted rate ratio, **HR**: hazard ratio,*

** Unadjusted estimates calculated from crude rates: as reported by Raguideau et al. [5]*

IX. References

1. Gatti M, Bianchin M, Raschi E, et al. Assessing the association between fluoroquinolones and emerging adverse drug reactions raised by regulatory agencies: An umbrella review. European Journal of Internal Medicine. 2020 May;75:60-70. doi: https://dx.doi.org/10.1016/j.ejim.2020.01.009. PubMed PMID: 31983604; English.

2. Yu X, Jiang DS, Wang J, et al. Fluoroquinolone Use and the Risk of Collagen-Associated Adverse Events: A Systematic Review and Meta-Analysis [Meta-Analysis

Research Support, Non-U.S. Gov't

Systematic Review]. Drug Safety. 2019 09;42(9):1025-1033. doi: https://dx.doi.org/10.1007/s40264-019-00828-z. PubMed PMID: 31077091; English.

3. Choi SY, Lim HA, Yim HW, et al. Administration of oral fluoroquinolone and the risk of rhegmatogenous retinal detachment: A nationwide population-based study in Korea [Research Support, Non-U.S. Gov't]. PLoS ONE [Electronic Resource]. 2018;13(4):e0195563. doi: https://dx.doi.org/10.1371/journal.pone.0195563. PubMed PMID: 29649310; English.

4. Alves C, Penedones A, Mendes D, et al. A systematic review and meta-analysis of the association between systemic fluoroquinolones and retinal detachment [Meta-Analysis

Review

Systematic Review]. Acta Ophthalmol (Oxf). 2016 Aug;94(5):e251-9. doi: https://dx.doi.org/10.1111/aos.12931. PubMed PMID: 26846201; English.

5. Raguideau F, Lemaitre M, Dray-Spira R, et al. Association Between Oral Fluoroquinolone Use and Retinal Detachment [Comparative Study]. JAMA Ophthalmol. 2016 Apr;134(4):415-21. doi: https://dx.doi.org/10.1001/jamaophthalmol.2015.6205. PubMed PMID: 26967005; English.

6. Chui CS, Wong IC, Wong LY, et al. Association between oral fluoroquinolone use and the development of retinal detachment: a systematic review and meta-analysis of observational studies [Meta-Analysis

Review

Systematic Review]. Journal of Antimicrobial Chemotherapy. 2015 Apr;70(4):971-8. doi: https://dx.doi.org/10.1093/jac/dku507. PubMed PMID: 25525200; English.

7. Eftekhari K, Ghodasra DH, Haynes K, et al. Risk of retinal tear or detachment with oral fluoroquinolone use: a cohort study [Comparative Study

Research Support, N.I.H., Extramural

Research Support, Non-U.S. Gov't]. Pharmacoepidemiology & Drug Safety. 2014 Jul;23(7):745-52. doi: https://dx.doi.org/10.1002/pds.3623. PubMed PMID: 24757075; English.

8. Fife D, Zhu V, Voss E, et al. Exposure to oral fluoroquinolones and the risk of retinal detachment: retrospective analyses of two large healthcare databases. Drug Safety. 2014 Mar;37(3):171-82. doi: https://dx.doi.org/10.1007/s40264-014-0138-y. PubMed PMID: 24526267; English.

9. Kapoor KG, Hodge DO, St Sauver JL, et al. Oral fluoroquinolones and the incidence of rhegmatogenous retinal detachment and symptomatic retinal breaks: a population-based study [Research Support, Non-U.S. Gov't]. Ophthalmology. 2014 Jun;121(6):1269-73. doi: https://dx.doi.org/10.1016/j.ophtha.2013.12.006. PubMed PMID: 24480710; English.

10. Kuo SC, Chen YT, Lee YT, et al. Association between recent use of fluoroquinolones and rhegmatogenous retinal detachment: a population-based cohort study [Observational Study

Research Support, Non-U.S. Gov't]. Clinical Infectious Diseases. 2014 Jan;58(2):197-203. doi: https://dx.doi.org/10.1093/cid/cit708. PubMed PMID: 24170197; English.

11. Pasternak B, Svanstrom H, Melbye M, et al. Association between oral fluoroquinolone use and retinal detachment [Research Support, Non-U.S. Gov't]. JAMA : the journal of the American Medical Association. 2013 Nov 27;310(20):2184-90. doi: https://dx.doi.org/10.1001/jama.2013.280500. PubMed PMID: 24281462; English.

12. Etminan M, Forooghian F, Brophy JM, et al. Oral fluoroquinolones and the risk of retinal detachment [Research Support, Non-U.S. Gov't]. JAMA : the journal of the American Medical Association. 2012 Apr 04;307(13):1414-9. doi: https://dx.doi.org/10.1001/jama.2012.383. PubMed PMID: 22474205; English.
